# Smoking and Chronic Obstructive Pulmonary Disease (COPD). Parallel Epidemics of the 21^st^ Century

**DOI:** 10.3390/ijerph6010209

**Published:** 2009-01-09

**Authors:** Rafael Laniado-Laborín

**Affiliations:** Facultad de Medicina, Universidad Autónoma de Baja Califonia, Mexico E-Mail: rafaellaniado@gmail.com; Tel.: 011-52-(664) 686-5626

**Keywords:** Tobacco, smoking, COPD, smoking cessation

## Abstract

One hundred million deaths were caused by tobacco in the 20^th^ century, and it is estimated that there will be up to one billion deaths attributed to tobacco use in the 21^st^ century. Chronic obstructive pulmonary disease (COPD) is rapidly becoming a global public health crisis with smoking being recognized as its most important causative factor. The most effective available treatment for COPD is smoking cessation. There is mounting evidence that the rate of progression of COPD can be reduced when patients at risk of developing the disease stop smoking, while lifelong smokers have a 50% probability of developing COPD during their lifetime. More significantly, there is also evidence that the risk of developing COPD falls by about half with smoking cessation. Several pharmacological interventions now exist to aid smokers in cessation; these include nicotine replacement therapy, bupropion, and varenicline. All pharmacotherapies for smoking cessation are more efficacious than placebo, with odds ratios of about 2. Pharmacologic therapy should be combined with nonpharmacologic (behavioral) therapy. Unfortunately, despite the documented efficacy of these agents, the absolute number of patients who are abstinent from smoking at 12 months of follow-up is low.

## 1. Introduction

**Figure 1:**
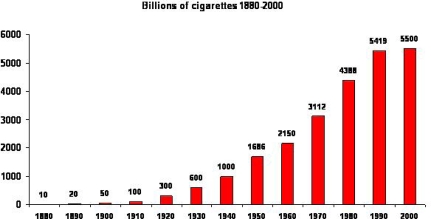
Global cigarette consumption. Increasing trend in the consumption of cigarettes (1880–2000)*. *Modified from Mackay, J.; Eriksen, M. *The Tobacco Atlas* © World Health Organization Publications.

According to the World Health Organization (WHO), one hundred million deaths were caused by tobacco in the 20^th^ century, and if current trends continue, there will be up to one billion deaths attributed to tobacco use in the 21^st^ century. There are more than one billion smokers in the world, and globally the use of tobacco products is increasing (Figure 1), with the epidemic shifting to the developing world. More than 80% of the world’s smokers live in low and middle income countries. It is estimated that tobacco use kills 5.4 million people a year and accounts for 10% of adult deaths worldwide, with up to 50% of smokers dying from a tobacco-use related disease. Unchecked, tobacco-related deaths will increase to more than eight million a year by 2030, and 80% of those deaths will occur in developing countries. Tobacco use is a risk factor for six of the eight leading causes of deaths in the world including respiratory and cardiovascular diseases, stroke and several malignant diseases [1].

Chronic obstructive pulmonary disease (COPD) is a major and growing cause of morbidity and mortality in countries at all levels of economic development [2, 3] with smoking being recognized as its most important causative factor [1, 3].

According to the WHO estimates, 80 million people in the world have moderate to severe COPD. More than 3 million people died of COPD in 2005, which corresponds to 5% of all adult deaths globally and it is estimated that by 2020 it will become the third leading cause of death worldwide [4]; this chronic disease is however, barely even acknowledged in the health statistics of many countries. Many patients remain undiagnosed, experience high levels of symptoms, their quality of life is often poor and they usually die prematurely of it or its complications [5–8].

Although cigarette smoking is the most commonly encountered tobacco-related risk factor for COPD [4], other types of tobacco smoking popular in various countries are also risk factors for COPD [9, 10] and air pollution resulting from the burning of wood and other biomass fuels, has also been identified as a COPD risk factor [4]. Passive exposure to cigarette smoke may also contribute to the development of COPD by increasing the lung total burden of inhaled particles and gases [11].

There is now evidence that most smokers develop some respiratory impairment due to COPD [12, 13]. One recent study addressing this issue [14] reported that 50% of smokers eventually develop COPD, as defined according to the Global Initiative for Chronic Obstructive Lung Disease (GOLD) guidelines [4]. This finding is of major clinical significance, in that it provides a scientific basis for the advice that can now be given to smokers that if they continue smoking lifelong, they have at least a one in two chances of developing COPD [12].

The costs of COPD to health services and society are substantial. Consultation rates in primary care are high and exacerbations of COPD are one of the most common causes of hospital admission. In developed countries, exacerbations of COPD account for the greatest burden on the health care system. In the European Union, the total direct costs of respiratory disease are estimated to be about 6% of the total health care budget, with COPD accounting for 56% (€38.6 billion) of this cost. In the United States in 2002, the direct costs of COPD were $18 billion and the indirect costs totaled $14.1 billion [4, 15].

## 2. Pathogenesis and Pathophysiology of Tobacco Induced Lug Injury

Cigarette smoke contains an extremely high concentration of oxidants. The reactive oxidant substances generated by smoking induce inflammation in the lung and its airway [16]; cigarette smoking causes an inflammatory process in the central airways, peripheral airways, and lung parenchyma, which is present even in smokers with normal lung function [17]. Studies have shown that in bronchial biopsies obtained from central airways, smokers have chronic inflammatory changes, with increased numbers of specific inflammatory cell types in different parts of the lung, and structural remodeling resulting from repeated injury and repair [18].

The exact role of smoking cessation on the airway inflammation process in COPD is still unknown. Cross-sectional studies suggest that there is still ongoing inflammation in COPD even after smoking cessation [19]. In general, the inflammatory and structural changes in the airways increase with disease severity and persist despite smoking cessation.

It is intriguing why airway inflammation is not resolved in COPD patients after 1 year of smoking abstinence, and even increases in some aspects. One explanation is that an inflammatory trigger persists after smoking cessation, keeping a local inflammatory response intact [20]. The persistent inflammation in chronic obstructive pulmonary disease may be, at least partly, related to repair of the smoke-induced tissue damage in the airways. It remains to be determined which parts of the observed inflammatory changes are beneficial and which harmful [20, 21]. A recently suggested hypothesis is that COPD may have an autoimmune component, which contributes to the airway inflammation even after smoking cessation [22]. These auto-antibodies may be directed against antigens present in tobacco or against endogenous auto-antigens that result from the smoking-induced inflammatory and oxidative lung injury [23].

## 3. Smoking Cessation and COPD

Smoking cessation is the single most effective—and cost-effective—treatment for COPD. Furthermore, smoking cessation is associated with a reduction in the risk of developing stroke, coronary heart disease, several types of cancer, and it is associated to an increased life expectancy [24].

Despite the ongoing inflammatory process, there is increasing evidence that the rate of development of COPD can be reduced when patients at risk of developing the disease stop smoking [25].

The first indications came from longitudinal cohort studies which showed that subjects who continued to smoke had a much steeper decline in lung function than those who had stopped smoking [26, 27].

The Lung Health Study confirmed that smoking cessation could reduce this smoking-related precipitous decline in lung function [28]. It was a randomized clinical trial designed to determine the potential benefits of smoking cessation. All participants had, as an inclusion criterion, asymptomatic airway obstruction. The special intervention participants received a smoking cessation program and were compared with usual care participants. Vital status was followed up to 14.5 years. The smoking intervention program consisted of a 10-week smoking cessation program that included a strong physician message and 12 group sessions using behavior modification and nicotine gum, plus either ipratropium or a placebo inhaler. At 5 years, 21.7% of special intervention participants had stopped smoking since study entry, compared with 5.4% of usual care participants. Smoking intervention participants had smaller declines in FEV-1 than usual care participants. Men who quit at the beginning of the study had an FEV-1 rate of decline of 30.2 mL/year, whereas women who quit declined at 21.5 mL/year. Men continuing to smoke throughout declined by 66.1 mL/year, and women continuing to smoke declined by 54.2 mL/year. More importantly, all-cause mortality was significantly lower in the special intervention group than in the usual care group (8.83 per 1000 person-years vs. 10.38 per 1000 person-years; p=0.03) [29].

It has been shown that repeated attempts to quit smoking, even with subsequent relapses, can prevent loss of lung function, especially in patients with mild COPD [30], and prolonged abstinence is also associated to a reduction in pulmonary symptoms [31].

If a smoker with advanced COPD smoking, he/she will not recover lost lung function, but the subsequent rate of decline is likely to revert towards normal [32]. Smoking cessation at an early stage of the disease has shown to improve prognosis [28, 32–33], and there is evidence that smoking cessation at an early stage of COPD is more effective than in the later stages [34].

Smokers seem to be intrinsically more motivated to stop smoking if they realize that their respiratory complaints are caused by smoking and that they are at risk of developing COPD [35]. In a recent study, smoking cessation rates were compared between smokers with COPD and smokers with normal lung function. During follow-up, the abstinence rates were significantly higher in smokers with COPD than in smokers with normal lung function [36].

## 4. Smoking Cessation Intervention Process

Tobacco dependence is a chronic condition that often requires repeated intervention to succeed [37]. Once users are dependent on tobacco, quitting is extremely difficult. Nicotine dependence resulting from tobacco use hinders efforts to sustain abstinence from tobacco for a prolonged period. Many users make multiple attempts to quit, often without the assistance that could significantly increase their chances of success. Studies have shown that a considerable number of smokers want to stop smoking, but a significant proportion of them have never tried [38]. A large proportion of all smokers are in the stage of contemplation or the stage of preparation. Most smokers go through several stages before they finally take the decision to make a cessation attempt and at last succeed with their intentions and stop smoking. Smoking cessation advice or other interventions appear to have their effect by triggering a cessation attempt. Each year about 2% of smokers succeed in quitting on their own initiative [39].

Smoking cessation is challenging and behavioral interventions alone have had only modest success; as a result drug therapy has been increasingly relied upon to assist in smoking cessation. The most common of these pharmacologic interventions has been nicotine replacement therapy (NRT) [40]. More recently, attention has focused on the use of anti-depressant therapy [4, 41]. Pharmacologic smoking cessation aids are recommended for all smokers trying to quit, unless contraindicated [42]; smokers should also be provided with counseling when attempting to quit [39]. Family physicians can play an important role in the smoking cessation process, given that 70% of smokers consult family physicians annually [43].

As mentioned, most smokers who attempt to quit do not utilize cessation aids, and as a result, they are usually unsuccessful with two-thirds relapsing within 48 hours [44]. For this reason, all smokers—including those who may be at risk for COPD as well as those who already have the disease — should be offered the most intensive smoking cessation intervention possible. Even a brief period of counseling at the doctor’s office to urge a smoker to quit, results in smoking cessation rates of 5 to 10% [45–47]. At the very least, this should be done for every smoker at every health care provider visit [48–51]. The rate of successful smoking cessation at one year is of 3–5% when the patient simply tries to stop, from 7–16% if the smoker undergoes behavioral intervention and up to 24% when receiving pharmacological treatment plus behavioral support. Recently, several meta-analyses have shown that all current pharmacotherapies for smoking cessation are twice as likely more efficacious than placebo [52–54].

Pharmacologic agents are classified as first-line pharmacotherapy, which includes nicotine replacement therapy, bupropion and varenicline and second-line agents such as nortriptyline, a tricyclic antidepressant agent, and clonidine, an antihypertensive drug [54].

## 5. Nicotine Replacement Therapy

Most regular smokers are addicted to nicotine. When a subject smokes a cigarette, nicotine will get into the bloodstream and almost immediately stimulate the brain. In regular smokers, when the blood level of nicotine decreases, withdrawal symptoms such as restlessness, increased appetite, inability to concentrate, irritability, dizziness, and nicotine craving will develop. These symptoms begin within a few hours after having the last inhalation of tobacco smoke; if they are not relieved by smoking a cigarette, withdrawal symptoms will increase in severity. Nicotine replacement therapies will allow the smoker to maintain stable nicotine levels in the bloodstream to avoid withdrawal symptoms without smoking.

Nicotine replacement therapies (NRTs) available include nicotine gum, patches, and inhalers. These therapies aid in smoking cessation by replacing the nicotine-mediated neuropharmacologic effects achieved by smoking [39]. Authors of a systematic review and meta-analysis which reviewed randomized trials of NRT compared with placebo or no treatment, and that had follow-up periods of 6 months or longer, determined that NRT doubles the likelihood of smoking cessation compared with no therapy (OR 1.77, 95% confidence interval [CI] 1.66–1.88) [40]. Evidence also indicates that the nicotine patch combined with another NRT is more effective than any single NRT [55, 56].

The potential side effects of nicotine gum include dyspepsia, singultus (hiccups) and a mandible pain. Nicotine patches are associated with cephalea, dizziness and nausea, sleep disturbances, and rash at the site of patch application. Adverse effects experienced by users of nicotine inhalers include throat and nasal irritation, rhinorrhea, sneezing, and coughing [57].

## 6. Bupropion

Bupropion was originally launched as a smoking cessation aid in 1997 and has now been approved in more than 50 countries [58]. It blocks the reuptake of dopamine and norepinephrine, which is thought to be the mechanism behind its effect on smoking cessation [59]. In a systematic review and meta-analysis of 31 bupropion trials in which bupropion was the sole agent used for cessation (compared with placebo or no pharmacotherapy), with 6 months follow-up or longer, the reviewers found the likelihood of cessation almost doubled with bupropion therapy [40, 60].

Bupropion alone or in combination with the nicotine patch has been found to significantly increase long-term cessation rates compared with the patch alone. The abstinence rates at 12 months were 15.6 percent in the placebo group, as compared with 16.4 percent in the nicotine-patch group, 30.3 percent in the bupropion group (P<0.001), and 35.5 percent in the group given bupropion and the nicotine patch (P<0.001). The greater abstinence rate with combination therapy (bupropion+nicotine patch) compared with bupropion alone, however, was not statistically significant [56].

The most common side effects associated with bupropion are dry mouth and insomnia. Additionally, the risk of seizures is thought to be 1 in 1000, and is associated with risk factors such as seizure disorders and eating disorders [61]. Only two seizures episodes were reported in the bupropion trials. However, the small number of observed seizures is likely due to the exclusion of patients at risk for seizures before randomization.

## 7. Varenicline

Varenicline is the first partial agonist of the α4β2 nicotinic acetylcholine receptor to be developed. The dependency effects of nicotine are thought to be mediated at these receptors [62, 63]. Varenicline works by stimulating dopamine, which results in reduced cravings and withdrawal symptoms. The drug also blocks nicotine receptors, which prevents the dopamine release associated with nicotine consumption [63, 64]. Varenicline might diminish withdrawal symptoms through an agonist effect and reduce craving through an antagonist effect; with nicotine exposure, the receptor occupancy of varenicline is expected to block the reinforcing effects of nicotine [65].

The most common side effect associated with varenicline is nausea. Other side effects include insomnia and abnormal dreaming [66, 67].

Recently, several trials have demonstrated the effectiveness of varenicline, in improving cessation rates [65, 66, 68–70]. Bupropion has been compared with varenicline in recent head-to-head randomized controlled trials [66, 68, 70]. These trials consistently favored varenicline. After pooling these data, it was found that rates of smoking abstinence associated with varenicline were about twice those associated with bupropion.

However, from the time of initial trials, varenicline has been associated with significant psychiatric adverse effects [71]. Recently the FDA issued two Med Watch advisories regarding reports of behavioral changes in patients taking varenicline. These have included erratic behavior, new onset of depressed mood, agitation, suicidal ideation, suicidal attempts, and completed suicides within days to weeks of initiating varenicline. Not all the individual affected had pre-existing histories of psychiatric illness [72]. There have been two recent published case reports of varenicline exacerbating symptoms of schizophrenia [73] and inducing a manic episode in a patient with bipolar disorder [74]. As mentioned, varenicline displaces nicotine from acetylcholine receptors, which in turn produces low to moderate levels of dopamine release, and stimulates the central nervous mesolimbic dopamine system. This may upset the balance in cholinergic-adrenergic tone, which has been implicated in the physiology of mania [71].

In summary varenicline seems to be the most effective of the available pharmacological agents for smoking cessation; however, the extensive exclusion criteria for most of these trials, the substantially different losses to follow up between groups, and the fact that most of these trial are funded by the industry might affect the trustworthiness and generalizability of these results [43].

Despite the early efficacy of these pharmacotherapies, the number of patients that remain abstinent from smoking at 1 year follow-up is low. Most of the randomized controlled trials report a point prevalence of abstinence at 12 months to be well under 30% among patients in the treatment groups. With continuous abstinence as the outcome measure, the rate of abstinence is even lower [64].

## 8. Nonpharmacologic Treatment

Nonpharmacologic cessation strategies include brief interventions, such as patient education and advice, behavioral therapy, self-help materials, and telephone counseling [65]. A review of randomized or quasi-randomized trials of individual behavioral counseling for smoking cessation by trained therapists with 6 months or longer follow-up indicated that individual counseling was more effective than no intervention at all. Counseling usually consists of one or more face-to-face sessions, often accompanied by telephone contact for support [66]. Evidence shows that group counseling is more effective than self-help and other less intensive intervention methods for smoking cessation [77]. It is unclear if group counseling is more effective than individual counseling, but it is more effective than no intervention [78]. Self-help materials might improve quit rates among smokers compared with those who receive no intervention, but the effect is generally small [79]. Proactive telephone counseling, in which the counselor initiates client contact, enhances the benefit of telephone counseling in comparison to reactive counseling, in which the client initiates contact. Multiple sessions of telephone counseling improve quit rates [80].

Web-based cessation programs have as well been found to be effective in smoking cessation [81, 82], however, more research is needed in this area [83].

## 9. Combined Therapy

Pharmacotherapy and counseling should be used in conjunction to further improve the chances of successful cessation [42]. Brief counseling combined with NRT has been found to be more effective than counseling alone in a randomized study of hospital patients receiving smoking cessation interventions [84, 85]. In a randomized study of telephone counseling combined with nicotine patch therapy, individuals who received both counseling and NRT had significantly greater abstinence rates compared with those who received NRT alone [86, 87].

How effective are the smoking cessation interventions in COPD patients? As mentioned the Lung Health Study (that included asymptomatic smokers with airway obstruction) reported that at 5 years, 21.7% of special intervention participants had stopped smoking since study entry, compared with 5.4% of usual care participants [28]. A recent Crochane review revealed the effectiveness of psychosocial interventions combined with pharmacological intervention compared to no treatment in COPD patients: psychosocial interventions combined with NRT versus no treatment increased the probability of success at a 5 year follow-up by four-fold (Relative risk= 4.19, 95% CI 3.41 to 5.15) [88].

## 10. Strategy for Smoking Cessation

Health care providers are key to the delivery of smoking cessation messages and interventions. Health care workers should encourage all patients who smoke to quit, even those patients who come to the health care provider for unrelated reasons and do not have symptoms of COPD, evidence of airflow limitation, or other smoking related disease.

The strategy for smoking cessation has utilized a mnemonic system based on the five “A’s” [4]:

1. **ASK:** Systematically identify all tobacco users at every visit. Write a patient’s smoking status in the medical chart under vital signs. Implement a system that ensures that, for every patient at every clinic visit, tobacco-use status is queried and documented. Ask patients about their desire to quit, and reinforce their intentions.
2. **ADVISE:** Strongly urge all tobacco users to quit. In a clear, strong, and personalized manner, urge every tobacco user to quit. Motivate patients who are reluctant to quit.
3. **ASSESS:** Most individuals go through several stages (precontemplation, contemplation, recycling) before they stop smoking. Determine willingness to make a quit attempt. Ask every tobacco user if he or she is willing to make a quit attempt at this time.
4. **ASSIST:** Aid the patient in quitting. Help the patient with a quit plan; help motivated smokers set a quit date. Provide practical counseling; provide, if available, intra-treatment social support; six first-line pharmacotherapies for tobacco dependence— bupropion SR, varenicline, nicotine gum, nicotine inhaler, nicotine nasal spray, and nicotine patch—are effective and at least one of these medications should be prescribed in the absence of contraindications.
5. **ARRANGE:** Schedule follow-up contact, either in person or via telephone. Encourage relapsed smokers to try quitting again. Tobacco dependence is a chronic condition that warrants repeated treatment until long-term or permanent abstinence is achieved.

There is limited data to guide clinicians in recommending one type of pharmacotherapy over another, the use of combinations, or how to match individual smokers to a particular form of therapy. Recently a Delphi approach was used to build consensus among a panel of international experts from various health disciplines with the purpose of developing decision rules for clinicians to guide differential prescribing practices and tailoring of pharmacotherapy for smoking cessation [89]. Factors that should be considered in prescribing pharmacotherapy include level of scientific evidence, patient preference and patient previous experience. The decision to prescribe combinations should be based on a history of failed attempts with monotherapy, patients with breakthrough cravings despite treatment and level of tobacco dependence. Other factors that should be taken into account on selection of pharmacotherapy include the potential impact on comorbidities and the presence of contraindications. There appears to be good justification for “off label” use such as higher doses of NRT or combination therapy in certain circumstances.

## 11. What can We Expect in the Near Future?

Although recent data suggest that current pharmacotherapy for smoking cessation is superior to placebo for treating nicotine dependence, a majority of smokers fail to maintain long-term abstinence from smoking. Thus, continued investigation of novel medications for nicotine dependence remains a critical priority. Promising agents include new non-nicotine-replacement pharmacotherapies [90–93] such as selegiline (a monoamine oxidase type B [MAO-B] inhibitor), fluoxetine (an antidepressant of the selective serotonin reuptake inhibitor [SSRI] class), naltrexone (an opioid receptor antagonist used primarily in the management of alcohol dependence and opioid dependence) and mecamylamine (a ganglionic blocker); other strategies include the development of a vaccine against nicotine dependence [94] and pharmacogenetic approaches to smoking cessation [95, 96].

## 12. Conclusions

Tobacco use kills more than five million people a year and accounts for 10% of adult deaths worldwide. COPD is a major and growing cause of morbidity and mortality with smoking being recognized as its most important causative factor. Several meta-analyses have shown that all pharmacotherapies for smoking cessation are twice as likely more efficacious than placebo with an abstinence rate in the 25–30% range at one year when pharmacological treatment and behavioral support are combined. Unfortunately, in spite of the initial efficacy of these pharmacotherapies, the number of patients that remain abstinent from smoking at 1 year follow-up is low (range 16–25%) [64].

## Figures and Tables

**Table 1:** Efficacy of pharmacologic treatment for smoking cessation *.

Treatment	Experimental group	Control Group	OR (CI95%)
Nicotine gum	19.5%	11.5%	1.66 (1.52, 1.81)
Nicotine patch	14.6%	8.6%	1.81 (1.63, 2.02)
Nicotine inhaler	17.15	9.1%	2.14 (1.44, 3.18)
Bupropion	20.0%	10.2%	1.94 (1.72, 2.19)
Varenicline	23.0%	10.3%	2.66 (1.72.4.11)

* Modified from Schmelzle, J., *et al*. [43]

## References

[1] http://www.who.int/topics/tobacco/facts/en/index.htlm (accessed 28 November 2008).

[2] Mannino DM, Buist AS (2007). Global burden of COPD: risk factors, prevalence, and future trends. Lancet.

[3] Buist AS, Vollmer WM, McBurnie MA (2008). Worldwide burden of COPD in high- and low-income countries. Part I. The Burden of Obstructive Lung Disease (BOLD) Initiative. Int. J. Tuberc. Lung Dis.

[4] *Global Strategy for the Diagnosis, Management and Prevention of COPD*. Global Initiative for Chronic Obstructive Lung Disease (GOLD) 2008.

[5] Menezes AM, Perez-Padilla R, Jardim JR, Muiño A, López MV, Valvidia G, Montes de Oca M, Talamo C, Hallal PC, Victoria CG, PLATINO team (2005). Chronic obstructive pulmonary disease in five Latin American cities (the PLATINO study): a prevalence study. Lancet.

[6] Halbert RJ, Natoli JL, Gano A, Badamgarav E, Buist AS, Mannino DM (2006). Global burden of COPD: systematic review and meta-analysis. Eur. Respir. J.

[7] Menezes AMB, Perez-Padilla R, Hallal PC, Jardim JR, Muiño A, Lopez MV, Valdivia G, Pertuze J, Montes de Oca M, Tálamo C, for the PLATINO Team (2008). Worldwide burden of COPD in high- and low-income countries. Part II. Burden of chronic obstructive lung disease in Latin America: the PLATINO study. Int. J. Tuberc. Lung Dis.

[8] Ko FWS, Hui DSC, Lai CKW (2008). Worldwide burden of COPD in high- and low-income countries. Part III. Asia-Pacific studies. Int. J. Tuberc. Lung Dis.

[9] Jindal SK, Aggarwal AN, Chaudhry K, Chhabra SK, D’Souza GA, Gupta D, Katiyar SK, Kumar R, Shah B, Vijayan VK (2006). A multicentric study on epidemiology of chronic obstructive pulmonary disease and its relationship with tobacco smoking and environmental tobacco smoke exposure. Indian J. Chest Dis. Allied Sci.

[10] Al-Fayez SF, Salleh M, Ardawi M, Azahran FM (1988). Effects of sheesha and cigarette smoking on pulmonary function of Saudi males and females. Trop. Geogr. Med.

[11] Eisner MD, Balmes J, Katz BP, Trupin L, Yelin E, Blanc P (2005). Lifetime environmental tobacco smoke exposure and the risk of chronic obstructive pulmonary disease. Environ. Health Perspect.

[12] Marsh S, Aldington S, Shirtcliffe P, Weatherall M, Beasley R (2006). Smoking and COPD: what really are the risks?. Eur. Respir. J..

[13] Rennard SI, Vestbo J (2006). COPD: the dangerous underestimate of 15%. Lancet.

[14] Lundback B, Lindberg A, Lindstrom M, Rönmark E, Jonsson AC, Jönsson E, Larsson LG, Andersson S, Sandström T, Larsson K (2003). Not 15 but 50% of smokers develop COPD? Report from the Obstructive Lung Disease in Northern Sweden Studies. Respir. Med.

[15] The ASPECT Consortium (2004). Tobacco on Health in the European Union: Past, present and future.

[16] Brody JS, Spira A (2006). Chronic Obstructive Pulmonary Disease, Inflammation, and Lung Cancer. Proc. Am. Thorac. Soc.

[17] Saetta M (1999). Airway Inflammation in Chronic Obstructive Pulmonary Disease. Am. J. Respir. Crit. Care Med.

[18] Saetta M, Di Stefano A, Maestrelli P, Ferraresso A, Drigo R, Potena A, Ciaccia A, Fabbri LM (1993). Activated T-lymphocytes and macrophages in bronchial mucosa of subjects with chronic bronchitis. Am. Rev. Respir. Dis.

[19] Rutgers SR, Postma DS, ten Hacken NH, Kauffman H, van der Mark TW, Koeter G, Timens W (2000). Ongoing airway inflammation in patients with COPD who do not currently smoke. Thorax.

[20] Willemse BWM, ten Hacken NHT, Rutgers B, Lesman-Leegte IGAT, Postma DS, Timens W (2005). Effect of 1-year smoking cessation on airway inflammation in COPD and asymptomatic smokers. Eur. Respir. J.

[21] Soriano JB, Agust A (2008). The yin and yang of COPD: or balancing repair (yang) and inflammation (yin). Eur. Respir. J.

[22] Agusti A, Macnee W, Donaldson K, Cosio M (2003). Hypothesis: does COPD have an autoimmune component?. Thorax.

[23] Cosio MG (2004). Autoimmunity, T-cells and STAT-4 in the pathogenesis of chronic obstructive pulmonary disease. Eur. Respir. J.

[24] Taylor DH, Hasselblad V, Henley SJ, Thun MJ, Sloan FA (2002). Benefits of smoking cessation for longevity. Am. J. Public Health.

[25] van Schayck CP, Kaper J (2006). Smoking and COPD: will they ever vanish into smoke? (Editorial). Primary Care Respir. J.

[26] Xu X, Dockery DW, Ware JH, Speizer FE, Ferris BG (1992). Effects of cigarette smoking on rate of loss of pulmonary function in adults: a longitudinal assessment. Am. Rev. Respir. Dis.

[27] Di Stefano A, Capelli A, Lusuardi M, Balbo P, Vecchio C, Maestrelli P, Mapp CE, Fabbri LM, Donner CF, Saetta M (1998). Severity of airflow limitation is associated with severity of airway inflammation in smokers. Am. J. Respir. Crit. Care Med.

[28] Anthonisen NR, Connett JE, Kiley JP, Altose MD, Bailey WC, Buist AS, Conway WA, Enright P, Kanner RE, O’Hara (1994). Effects of smoking intervention and the use of an inhaled anticholinergic bronchodilator on the rate of decline of FEV 1. The Lung Health Study. JAMA.

[29] Anthonisen NR, Skeans MA, Wise RA, Manfreda J, Kanner RE, Connett JE (2005). The Effects of a Smoking Cessation Intervention on 14.5-Year Mortality.A Randomized Clinical Trial. Ann. Intern. Med.

[30] Murray RP, Anthonissen NR, Connett JE, Wise RA, Lindgren PG, Greene PG, Nides MA (1998). Effect of multiple attempts to quit smoking and relapses to smoking on pulmonary function. J. Clin. Epid.

[31] Kanner RE, Connett JE, Williams DE, Buist AS (1999). Effects of randomized assignment to a smoking cessation intervention and changes in smoking habits on respiratory symptoms in smokers with mild chronic obstructive pulmonary disease: The Lung Health Study. Am. J. Med.

[32] Fletcher C, Peto R, Tinker C (1977). The natural history of chronic airflow obstruction. BMJ.

[33] Xu X, Dockery DW, Ware JH, Speizer FE, Ferris BG (1992). Effects of cigarette smoking on rate of loss of pulmonary function in adults: a longitudinal assessment. Am. Rev. Respir. Dis.

[34] Wagena EJ, Knipschild P, Huibers MJH, Wouters EFM, van Schayck CP (2005). The efficacy of bupropion and nortriptyline for smoking cessation among people who are at risk for or have chronic obstructive pulmonary disease: a randomizaed, placebo-controlled trial. Arch. Int. Med.

[35] Zielinszky J, Bednarek M (2001). Early detection of COPD in a high-risk population using spirometric screening. Chest.

[36] Stratelis G, Mölstad S, Jakobsson P, Zetterström O (2006). The impact of repeated spirometry and smoking cessation advice on smokers with mild COPD. Scandinavian. J. Primary Health Care.

[37] Anderson JE, Jorenby DE, Scott WJ, Fiore MC (2002). Treating tobacco use and dependence: An evidence-based clinical practice guideline for tobacco cessation. Chest.

[38] Jimenez-Ruiz CA, Masa F, Miravitlles M, Gabriel R, Viejo JL, Villasante C, Sobradillo V (2001). Smoking characteristics: Differences in attitudes and dependence between healthy smokers and smokers with COPD. Chest.

[39] Hughes JR (2003). Motivating and helping smokers to stop smoking. J. Gen. Intern. Med.

[40] Silagy C, Lancaster T, Stead L, Mant D, Fowler G (2004). Nicotine replacement therapy for smoking cessation. Cochrane Database Syst. Rev..

[41] Hughes J, Stead L, Lancaster T (2004). Antidepressants for smoking cessation. Cochrane Database Syst. Rev..

[42] The Tobacco Use and Dependence Clinical Practice Guideline Panel, Staff, and Consortium Representatives (2000). A clinical practice guideline for treating tobacco use and dependence: a US Public Health Service report. JAMA.

[43] Schmelzle J, Rosser WW, Birtwhistle R (2008). Update on pharmacologic and nonpharmacologic therapies for smoking cessation. Can. Fam. Physician.

[44] Hughes JR, Gulliver SB, Fenwick JW, Valliere WA, Cruser K, Pepper S, Shea P, Solomon LJ, Flynn BS (1992). Smoking cessation among self-quitters. Health Psychol.

[45] Morgan MD, Britton JR (2003). Chronic obstructive pulmonary disease 8: Non-pharmacological management of COPD. Thorax.

[46] Wu P Wilson K Dimoulas P Mills EJ Effectiveness of smoking cessation therapies: a systematic review and meta-analysis BMC Public Health 2006 6 300doi: 10.1186/1471-2458-6-300. 17156479 10.1186/1471-2458-6-300 PMC1764891

[47] Lancaster T, Stead LF (2004). Physician advice for smoking cessation. Cochrane Database Syst. Rev..

[48] Britton J, Knox A (1999). Helping people to stop smoking: the new smoking cessation guidelines. Thorax.

[49] Fiore MC, Bailey WC, Cohen SJ, Dorfman SF, Fox BJ, Goldstein MG, Gritz E, Hasselblad V, Heyman RB, Jaen CR (2000). The Tobacco Use and Dependence Clinical Practice Guideline Panel, Staff, and Consortium Representatives. A clinical practice guideline for treating tobacco use and dependence: A US Public Health Service report. JAMA.

[50] Pauwels RA, Buist AS, Ma P, Jenkins CR, Hurd SS (2001). Global strategy for the diagnosis, management, and prevention of chronic obstructive pulmonary disease: National Heart, Lung, and Blood Institute and World Health Organization Global Initiative for Chronic Obstructive Lung Disease (GOLD): executive summary. Am. J. Respir. Crit. CareMed.

[51] van Schayck CP, Kaper J (2006). Smoking and COPD: will they ever vanish into smoke? (Editorial). Primary Care Respir. J.

[52] Wu P Wilson K Dimoulas P Mills EJ Effectiveness of smoking cessation therapies: a systematic review and meta-analysis BMC Public Health 2006 6 300doi:10.1186/1471-2458-6-300. 17156479 10.1186/1471-2458-6-300 PMC1764891

[53] Ranney L, Melvin C, Lux L, McClain EMA, Lohr KN (2006). Systematic Review: Smoking Cessation Intervention Strategies for Adults and Adults in Special Populations. Ann. Intern. Med.

[54] Nides M (2008). Update on pharmacologic options for smoking cessation treatment. Am. J. Med.

[55] Stead LF, Perera R, Bullen C, Mant D, Lancaster T (2008). Nicotine replacement therapy for smoking cessation. Cochrane Database Syst. Rev.

[56] Jorenby DE, Leischow SJ, Nides MA, Rennard SI, Johnston JA, Hughes AR, Smith SS, Muramoto ML, Daughton DM, Doan K, Fiore MC, Baker TB (1999). A controlled trial of sustained-release bupropion, a nicotine patch, or both for smoking cessation. N. Engl. J. Med.

[57] McClure JB, Swan GE (2006). Tailoring nicotine replacement therapy: rationale and potential approaches. CNS Drugs.

[58] Aubin HJ (2002). Tolerability and safety of sustained-release bupropion in the management of smoking cessation. Drugs.

[59] Hays JT, Ebbert JO (2003). Bupropion for the treatment of tobacco dependence: guidelines for balancing risks and benefits. CNS Drugs.

[60] Hughes JR, Stead LF, Lancaster T (2007). Antidepressants for smoking cessation. Cochrane Database Syst. Rev.

[61] Ferry L, Johnston JA (2003). Efficacy and safety of bupropion SR for smoking cessation: data from clinical trials and five years of postmarketing experience. Int. J. Clin. Pract.

[62] Coe JW, Brooks PR, Vetelino MG, Wirtz MC, Arnold EP, Huang J, Sands SB, Davis TI, Lebel LA, Fox CB, Shrikhande A, Heym JH, Schaeffer E, Rollema H, Lu Y, Mansbach RS, Chambers LK, Rovetti CC, Schulz DW, Tingley FD, O’Neill BT (2005). Varenicline: an α4b2 nicotinic receptor partial agonist for smoking cessation. J. Med. Chem.

[63] Foulds J (2006). The neurobiological basis for partial agonist treatment of nicotine dependence: varenicline. Int. J. Clin. Pract.

[64] Eisenberg MJ, Filion KB, Yavin D, Bélisle P, Mottillo S, Joseph L, Gervais A, O’Loughlin J, Paradis G, Rinfret S, Pilote L (2008). Pharmacotherapies for smoking cessation: a meta-analysis of randomized controlled trials. CMAJ.

[65] Oncken C, Gonzales D, Nides M, Rennard S, Watsky E, Billing CB, Anziano R, Reeves K (2006). Efficacy and safety of the novel selective nicotinic acetylcholine receptor partial agonist, varenicline, for smoking cessation. Arch. Intern. Med.

[66] Jorenby DE, Hays JT, Rigotti NA, Azoulay S, Watsky EJ, Williams KE, Billing CB, Gong J, Reeves KR (2006). Efficacy of varenicline, an α4b2 nicotinic acetylcholine receptor partial agonist, vs. placebo or sustained-release bupropion for smoking cessation: a randomized controlled trial. JAMA.

[67] Gonzales D, Rennard SI, Nides M, Oncken C, Azoulay S, Billing CB, Watsky EJ, Gong J, Williams KE, Reeves KR (2006). Varenicline, an α4b2 nicotinic acetylcholine receptor partial agonist, vs. sustained-release bupropion and placebo for smoking cessation: a randomized controlled trial. JAMA.

[68] Tonstad S, Tonnesen P, Hajek P, Williams KE, Billing CB, Reeves KR (2006). Effect of maintenance therapy with varenicline on smoking cessation: a randomized controlled trial. JAMA.

[69] Nides MCO, Gonzales D, Rennard SI, Watsky EJ, Anziano R, Reeves KR (2006). Smoking cessation with varenicline, a selective alpha-4-beta-2 nicotinic receptor partial agonist: results from a 7-week, randomized, placebo- and bupropion controlled trial with 1-year follow-up. Arch. Intern. Med.

[70] Lyon GJ (2008). Possible varenicline-induced paranoia and irritability in a patient with major depressive disorder, borderline personality disorder, and methamphetamine abuse in remission. J. Clin. Psychopharmacol.

[71] Pumariega AJ, Nelson R, Rotenberg L (2008). Varenicline-Induced Mixed Mood and Psychotic Episode in a Patient with a Past History of Depression. CNS Spectr.

[72] Food and Drug Administration www.fda.gov/cder/drug/infopage/varenicline/default.htm.

[73] Kohen I, Kremen N (2007). Varenicline-induced manic episode in a patient with bipolar disorder. Am. J. Psychiatry.

[74] Freedman R (2007). Exacerbation of schizophrenia by varenicline [letter]. Am. J. Psychiatry.

[75] Schmelzle J, Rosser WW, Birtwhistle R (2008). Update on pharmacologic and nnpharmacologic therapies for smoking cessation. Can. Fam. Physician.

[76] Sutherland G (2002). Current approaches to the management of smoking cessation. Drugs.

[77] Lancaster T, Stead LF (2005). Individual behavioral counseling for smoking cessation. Cochrane Database Syst. Rev.

[78] Willemse B, Lesman-Leegte I, Timens W, Postma D, ten Hacken N (2005). High Cessation Rates of Cigarette Smoking in Subjects With and Without COPD. Chest.

[79] Stead LF, Lancaster T (2005). Group behaviour therapy programmes for smoking cessation. Cochrane Database Syst. Rev.

[80] Lancaster T, Stead LF (2005). Self-help interventions for smoking cessation. Cochrane Database Syst. Rev.

[81] Stead LF, Perera R, Lancaster T (2006). Telephone counseling for smoking cessation. Cochrane Database Syst. Rev.

[82] Swartz LH, Noell JW, Schroeder SW, Ary DV (2006). A randomised control study of a fully automated internet based smoking cessation programme. Tob. Control.

[83] Strecher VJ, Shiffman S, West R (2005). Randomized controlled trial of a webbased computer-tailored smoking cessation program as a supplement to nicotine patch therapy. Addiction.

[84] Etter JF (2006). Internet-based smoking cessation programs. Int. J. Med. Inform.

[85] Molyneux A, Lewis S, Leivers U, Anderton A, Antoniak M, Brackenridge A, Nilsson F, McNeill A, West R, Moxham J, Britton J (2003). Clinical trial comparing nicotine replacement therapy (NRT) plus brief counseling, brief counseling alone, and minimal intervention on smoking cessation in hospital inpatients. Thorax.

[86] Simon JA, Carmody TP, Hudes ES, Snyder E, Murray J (2003). Intensive smoking cessation counselling versus minimal counselling among hospitalized smokers treated with transdermal nicotine replacement: a randomized trial. Am. J. Med.

[87] Macleod ZR, Charles MA, Arnaldi VC, Adams IM (2003). Telephone counselling as an adjunct to nicotine patches in smoking cessation: a randomised controlled trial. Med. J. Aust.

[88] Bader P McDonald PW Selby P An Algorithm for Tailoring Pharmacotherapy for Smoking Cessation: Results from a Delphi Panel of International Experts Tob. Control 2008 13[Epub ahead of print doi:10.1136/tc.2008.025635. 10.1136/tc.2008.025635 PMC2614465 18845621

[89] van der Meer RM, Wagena EJ, Ostelo RW, Jacobs JE, van Schayck CP (2003). Smoking cessation for chronic obstructive pulmonary disease. Database Syst. Rev.

[90] Halpin DMG, Miravitlles M (2006). Chronic Obstructive Pulmonary Disease. The Disease and Its Burden to Society. Proc. Am. Thorac. Soc.

[91] Siu EC, Tyndale RF (2008). Selegiline is a mechanism-based inactivator of CYP2A6 inhibiting nicotine metabolism in humans and mice. J. Pharmacol. Exp. Ther.

[92] Schnoll RA, Lerman C (2006). Current and emerging pharmacotherapies for treating tobacco dependence. Expert Opin. Emerg. Drugs.

[93] Foulds J, Steinberg MB, Williams JM, Ziedonis DM (2006). Developments in pharmacotherapy for tobacco dependence: past, present and future. Drug Alcohol Rev.

[94] Maurer P, Jennings GT, Willers J, Rohner F, Lindman Y, Roubicek K, Renner WA, Müller P, Bachmann MF (2005). A therapeutic vaccine for nicotine dependence: preclinical efficacy, and phase I safety and immunogenicity. Eur. J. Immunol.

[95] Berrettini WH, Lerman CE (2005). Pharmacotherapy and pharmacogenetics of nicotine dependence. Am. J. Psychiatry.

[96] Pride NB (2001). Smoking cessation: effects on symptoms, spirometry and future trends in COPD. Thorax.

